# An Overview of the Recent Development of Anticancer Agents Targeting the HIF-1 Transcription Factor

**DOI:** 10.3390/cancers13112813

**Published:** 2021-06-04

**Authors:** Yukari Shirai, Christalle C. T. Chow, Gouki Kambe, Tatsuya Suwa, Minoru Kobayashi, Itsuki Takahashi, Hiroshi Harada, Jin-Min Nam

**Affiliations:** 1 Laboratory of Cancer Cell Biology, Graduate School of Biostudies, Kyoto University, Yoshida-Konoe-Cho, Sakyo-Ku, Kyoto 606-8501, Japan; shirai.yukari.52c@st.kyoto-u.ac.jp (Y.S.); chow.tung.85r@st.kyoto-u.ac.jp (C.C.T.C.); kambe.gouki.83z@st.kyoto-u.ac.jp (G.K.); tsuwa@kuhp.kyoto-u.ac.jp (T.S.); kobayashi.minoru.4m@kyoto-u.ac.jp (M.K.); takahashi.itsuki.35x@st.kyoto-u.ac.jp (I.T.); 2 Department of Genome Repair Dynamics, Radiation Biology Center, Graduate School of Biostudies, Kyoto University, Yoshida-Konoe-Cho, Sakyo-Ku, Kyoto 606-8501, Japan

**Keywords:** cancer, tumor hypoxia, hypoxia-inducible factor 1 (HIF-1), HIF-1 inhibitor, chemoresistance, radioresistance

## Abstract

**Simple Summary:**

As hypoxia-inducible factor 1 (HIF-1) is recognized as a target for cancer therapy, extensive efforts have been devoted to developing its inhibitors. We provide an overview of the latest information about basic, translational, and clinical research for the development of HIF-1 inhibitors.

**Abstract:**

Hypoxia, a characteristic feature of solid tumors, is associated with the malignant phenotype and therapy resistance of cancers. Hypoxia-inducible factor 1 (HIF-1), which is responsible for the metazoan adaptive response to hypoxia, has been recognized as a rational target for cancer therapy due to its critical functions in hypoxic regions. In order to efficiently inhibit its activity, extensive efforts have been made to elucidate the molecular mechanism underlying the activation of HIF-1. Here, we provide an overview of relevant research, particularly on a series of HIF-1 activators identified so far and the development of anticancer drugs targeting them.

## 1. Introduction

Malignant solid tumors grow rapidly with a formation of low oxygen regions below physiological levels. Tumor hypoxia is caused by an imbalance between oxygen supply to and oxygen consumption in cancer cells, for multiple reasons, such as the relatively slower formation rate of tumor vasculature compared to tumor growth [1,2,3,4]. Cancer cells located in the distal regions of tumor vasculatures cannot get enough oxygen because of the oxygen consumption by cancer cells surrounding vasculatures, causing hypoxic regions in malignant solid tumors [1,2,3,4]. Tortuous and leaky architecture of tumor vasculature is also one of the causes of tumor hypoxia [1,2,3,4]. Hypoxia leads to the malignant phenotype and therapeutic resistance of cancer. [1,2,3,4]. It has been revealed that cellular responses to hypoxia are regulated by some factors, but hypoxia-inducible factor 1 (HIF-1), which induces the transcription of various genes related to angiogenesis, glucose metabolism, cell proliferation, survival, invasion, and metastasis, is recognized as a master regulator of the hypoxia response [5,6,7]. The HIF-1 pathway is an attractive target to prevent cancer aggressiveness and improve the effectiveness of cancer therapy.

**Figure 1:**
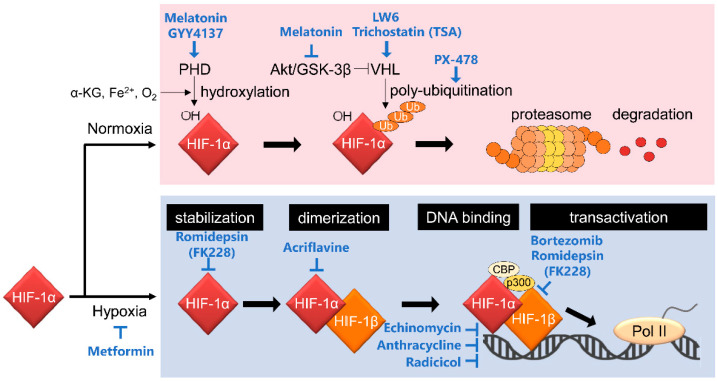
Canonical regulatory mechanisms behind the activation of HIF-1 and HIF-1 inhibitors targeting them. HIF-1 activity is mainly regulated at the HIF-1α protein stability level through PHD-dependent hydroxylation and VHL-mediated ubiquitination. See the main text for details.

HIF-1 is a heterodimeric transcription factor that comprises HIF-1α and HIF-1β subunits (Figure 1). HIF-1α expression is influenced by oxygen levels surrounding cells and is induced under hypoxic conditions. In contrast, the HIF-1β subunit, also known as aryl hydrocarbon receptor nuclear translocator (ARNT), is constitutively expressed regardless of oxygen conditions [8]. These two subunits have functional domains, basic helix–loop–helix (bHLH) and Per-Arnt-Sim (PAS) domains, enabling hetero-dimerization and binding to the enhancer region, hypoxia response element (HRE), on its target gene loci. Importantly, HIF-1α, but not HIF-1β, has an oxygen-dependent degradation (ODD) domain and COOH-terminal transactivation domains (C-TAD). The ODD domain plays an important role in oxygen-regulated HIF-1α degradation. Under normoxic conditions, with a normal level of oxygen, proline residues at positions 402 and 564 in the ODD domain are hydroxylated by proryl-4-hydroxylases (PHDs) [9,10]. The hydroxylated HIF-1α is ubiquitinated by E3 ubiquitin ligase containing the von Hippel–Lindau tumor suppressor protein (pVHL) for proteolysis by the 26S proteasome [11,12,13]. The asparagine residue positioned at 803 in C-TAD of HIF-1α is hydroxylated by factor inhibiting HIF-1 (FIH-1) under normoxia. The oxygen-dependent hydroxylation inhibits recruitment of the transcriptional coactivator CREB-binding protein (CBP)/p300 to C-TAD, and it leads to a reduction of HIF-1 transcriptional activity [14,15,16].

Both PHDs and FIH-1 are categorized as members of α-ketoglutarate (α-KG)-dependent dioxygenase (α-KGDD) family proteins, which need not only oxygen but also α-KG and Fe^2+^ for their enzymatic activity. Therefore, HIF-1α protein becomes unstable not only when oxygen becomes unavailable but also when the levels of either α-KG or Fe^2+^ decrease even in the presence of oxygen. Genetic mutations and metabolic disturbances which decrease the intracellular levels of α-KG have been reported to stabilize the HIF-1α protein. Since α-KG is an intermediate metabolite in the Krebs/trichloroacetic acid (TCA) cycle, functional disorder in its key components, fumarate hydratase (FH), succinate dehydrogenase (SDH), and isocitrate dehydrogenase (IDH), influence HIF-1 activity. Loss-of-function mutations in SDH and FH cause accumulation of succinate and fumarate, respectively, which inhibits α-KGDD through product inhibition and eventually activates HIF-1 [17,18]. Mutations in IDH1/2 and aberrant overexpression of IDH3 have also been reported to activate HIF-1 through the production of 2-hydroxyglutarate (2-HG) and the decrease in α-KG levels, respectively [19,20,21,22]. In addition to the metabolic disturbances, an increase in the intracellular ROS levels, for example, hydrogen peroxide (H_2_O_2_), is also known to stabilize HIF-1α protein by a decrease in Fe^2+^, such as through the Fenton reaction (Fe^2+^ + H_2_O_2_ + -> Fe^3+^ + OH^−^ + .OH) [21,23,24].

**Figure 2:**
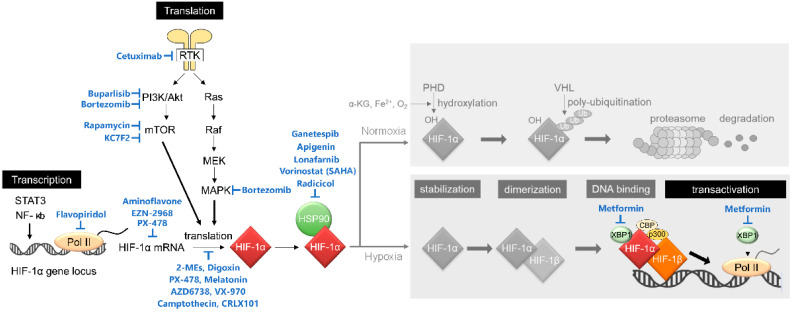
Non-canonical regulatory mechanisms behind the activation of HIF-1. HIF-1 activity is regulated at multiple steps, including transcriptional initiation of the HIF-1α gene, translational initiation of HIF-1α mRNA, stability of HIF-1α protein, and transactivation of HIF-1α. See the main text for details.

Hence, HIF-1 activity is known to be mainly regulated through post-translational modification by PHDs and FIH-1, but accumulating evidence has revealed that it is also regulated at other steps including transcriptional initiation, translational initiation, protein–protein interaction, and post-translational modification by other factors (Figure 2). For example, transcription of the HIF-1α gene is upregulated through the binding of the ISGF3 complex (composed of ATAT1, STAT2, and IRF9), STAT3, or NF-κB to the promoter region of HIF-1α locus [25,26,27], through the activation of the PI3K/Akt/PKC/HDAC pathway [28], or through inactivation of PTEN by aberrant activation of LY6E [29]. Translational initiation of HIF-1α is known to be upregulated by the PI3K/Akt pathway [30,31,32]. The stability of the HIF-1α protein is regulated not only by the PHDs-VHL-dependent manner described above but also by HSP90 and UCHL1; HSP90 protects HIF-1α from RACK1-mediated and PHDs-VHL-independent proteolysis [33], and, on the other hand, UCHL1 deubiquitinates HIF-1α protein for its stabilization [34].

In this review, we will provide a brief overview of the regulatory mechanisms of HIF-1 in cancer and focus on anticancer drugs that could target HIF-1α expression and HIF-1 activity (Table 1 and Table 2).

## 2. Mechanisms of Action of HIF-1 Inhibitors

### 2.1. Inhibitors of Transcriptional Initiation of HIF-1α

Transcriptional initiation is one of the important regulatory steps that influences HIF-1α mRNA expression levels. It is stimulated by the binding of transcription factors, such as signal transducer and activator of transcription 3 (STAT3) and nuclear factor kappa-light-chain-enhancer of activated B cells (NF-κB), to the promoter region of the HIF-1α gene locus [27,122] (Figure 1).

- Flavopiridol

Flavopiridol (alvocidib), a kind of synthetic flavonoid derived from an alkaloid in *Amoora robituka* and *Dysoxylum binectariferum* (Indian plant), is known as a cyclin-dependent kinase (CDK) inhibitor [123]. The drug inhibits RNA polymerase II-dependent transcription of mRNA by blocking the phosphorylation of its COOH-terminal domain, and thereby prevents global transcription including HIF-1α [35,36,37], leading to anti-tumor effects, confirmed in a clinical study [38,39]. The effect of flavopiridol was tested in a phase II clinical trial for patients with acute myeloid leukemia; however, there were no significant differences in overall survival despite higher complete remission rates [124]. Currently, dinaciclib, a second-generation CDK inhibitor, is undergoing a phase III trial. Dinaciclib shows fewer off-target effects than flavopiridol in patients with chronic lymphocytic leukemia. However, the effect of dinaciclib on HIF-1 has not yet been demonstrated.

Importantly, in addition to flavopiridol, there are numerous flavonoids, such as quercetin, epigallocatechin-3-gallate (EGC), deguelin, baicalein, chrysin, kaempferol, apigenin, bavachinin, alpinumisoflavone (ALP), naringin, oroxylin A, and wogonin [125]. The anti-cancer effects of flavonoids are mediated, in part, by a decrease in HIF-1 activity [125]. In vitro studies suggest that flavonoids might exhibit favorable anti-cancer activity, and further investigations are required to develop them as cancer drugs.

- Aminoflavone (AF)

Aryl hydrocarbon receptor (AhR), as well as HIF-1α, dimerizes with HIF-1β and acts as a transcription factor by binding to the xenobiotic response element (XRE) in the promoter regions of its downstream target genes. There is a competitive crosstalk between AhR and HIF-1 pathways; the activity of HIF-1 is inhibited by the AhR pathway [126,127]; therefore, aminoflavone, a ligand of AhR, is expected to inhibit HIF-1. Surprisingly, aminoflavone was reported to almost completely suppress the accumulation and transactivation activity of HIF-1α protein [40]. Although the molecular mechanism behind the inhibitory effect was not clearly understood, surprisingly aminoflavone was demonstrated to inhibit transcriptional initiation of the HIF-1α gene even in the functional AhR-deficient cells. Regardless of whether the inhibitory effect of aminoflavone is independent of AhR or not, it showed anticancer effects in preclinical experiments using an MCF-7 xenografted tumor model, for example, and in patients with breast and renal cancers [41,42,43]. Aminoflavone has been tested in several clinical trials; however, these clinical trials have been terminated or the results have been withdrawn, leading to unproven clinical utility.

### 2.2. Inhibitor of HIF-1α mRNA Stabilization

- EZN-2968

Antisense oligonucleotide treatment is recognized as a potential strategy that can specifically inhibit target mRNA expression. EZN-2968, a third-generation antisense oligonucleotide, has been developed to hybridize with HIF-1α mRNA. EZN-2968 treatment decreased mRNA and protein levels of HIF-1α under hypoxic conditions in a dose-dependent manner and significantly delayed tumor growth in DU145 xenografted tumor-bearing mice [44]. Recently, phase I clinical trials demonstrated its effectiveness in reducing HIF-1α expression and improving the clinical response with acceptable toxicity in patients with a wide variety of solid tumors [45,128,129]. Further evaluation will be needed in a phase II trial with more patients.

### 2.3. Inhibitors of Translational Initiation of HIF-1α

#### 2.3.1. Targeting the RTK/PI3K/Akt/mTOR Pathway

The receptor tyrosine kinase (RTK)/phosphatidylinositol-3-kinase (PI3K)/Akt (also known as protein kinase B)/mammalian target of rapamycin (mTOR) pathway has been reported to regulate the translational initiation of HIF-1α mRNA [31,46,130].

- Rapamycin

The 5′-UTR in HIF-1α mRNA contains the 5′-terminal oligopolypyrimidine tract (5′-TOP), a key component to receive the regulation of translational initiation by mTOR and its downstream substrates [31]. Several reports have suggested that aberrantly upregulated mTOR signaling correlates with elevated HIF-1α expression. Therefore, the inhibition of mTOR signaling suppresses HIF-1α mRNA translation and cell proliferation [47,48,131]. Each mTOR inhibitor, rapamycin, and its derivatives, temsirolimus, and everolimus, form a ternary complex with FK506-bonding protein 12 (FKBP12) and mTOR and physically inhibits the interaction between mTOR and its phosphorylation substrate protein. These reagents exhibited favorable antitumor effects in animal studies using, e.g., the ACHN or SN12C tumor xenograft model and are currently approved and applied for the treatment of various solid tumors [46,47,48,132].

- Cetuximab

mTOR activity is regulated by its upstream signaling pathway, composed of receptor tyrosine kinases (RTKs) and PI3K/Akt. Consistent with mTOR inhibition, RTK inhibitors suppress the expression of HIF-1α [133]. For example, an epidermal growth factor receptor (EGFR) inhibitor, cetuximab, which is approved by the FDA, suppresses the expression of HIF-1α and sensitizes head and neck squamous cell carcinoma (HNSCC) cells to radiotherapy [49]. An anntitumor effect was confirmed in various animal models using *tgfb1*/*pten* deletion 2cKO mouse and CAL27 tumor xenograft, etc. [49,50].

- Buparlisib

PI3K inhibitors, LY294002 and wortmannin, have been confirmed to suppress HIF-1 protein synthesis in various types of cancer cell lines [134]. While LY294002 and wortmannin did not reach clinical trials due to their toxicity, buparlisib, an ATP competitive pan-PI3K inhibitor, first underwent phase III clinical trials. Patients with advanced breast cancer received buparlisib in combination with a 7α-alkylsulphinyl analogue of 17β-estradiol, fulvestrant. Although a combined regimen led to higher frequencies of adverse events, buparlisib prolonged progression-free survival compared with fulvestrant alone [51,135].

- KC7F2

KC7F2 is a cystamine compound which was identified as a potent inhibitor of HIF-1α through an HIF-responsive reporter-based screening [52]. KC7F2 suppresses HIF-1α protein synthesis in cancer cell lines U251MG, MCF7, PC3, and LNZ308 under hypoxic conditions. Mechanistically, KC7F2 targets the mTOR complex 1 pathway, 4E binding protein 1, and p70 S6 kinase, which leads to the inhibition of HIF-1α protein synthesis. Treatment with KC7F does not affect the transcription of HIF-1α mRNA or the stability of HIF-1α protein. It is worth noting that KC7F2 inhibits transcriptions of HIF-1 target genes, such as carbonic anhydrase IX (CAIX), matrix metalloprotease 2 (MMP-2), enolase 1, and endothelin 1 in LN229 cells under hypoxic conditions [52]. KC7F2 has not been evaluated in clinical trials.

#### 2.3.2. Inhibitors of Microtubule Dynamics and Na+/K+ ATPase

- 2-MEs

Translational initiation of HIF-1α mRNA is also regulated by microtubule dynamics. Several lines of reports have indicated that the disruption of microtubule dynamics suppresses HIF-1α mRNA translation and leads to the accumulation of HIF-1α mRNA into P-bodies. A derivative of estradiol with no estrogenic activity, 2-methoxyestradiol (2-ME), inhibits microtubule polymerization by binding to tubulin; therefore, it is categorized as a microtubule-targeting agent. 2-ME and its derivatives were shown to inhibit HIF-1α mRNA translation and exhibit anti-tumor activity in vitro and with the use of, e.g., LLC and MDA-MB-231 xenografted tumor models with in vivo [53,54,55]. In contrast to previous in vitro and in vivo studies, there were no objective responses in phase II clinical trials [56,57].

- Digoxin

Cardiac glycosides, such as digoxin, ouabain, and proscillaridin A, are organic compounds that inhibit Na+/K+ ATPase. Digoxin inhibits HIF-1α protein synthesis, leading to a decrease in tumor growth in xenografts [58]. Although the mechanism of action of cardiac glycosides is unclear, digoxin treatment suppresses neovascularization by decreasing HIF-1α levels in P493 and PC3 tumor xenografts in vivo [59]. Digoxin has passed phase II clinical trials.

#### 2.3.3. Translational Regulation of HIF-1α by ATR

- AZD6738 and VX-970

A serine/threonine kinase, ataxia telangiectasia and Rad3-related kinase (ATR), belongs to the phosphoinositide 3-kinase-related kinase (PIKK) family and is involved in DNA damage response signaling. Replicative stress induced by hypoxia leads to the activation of ATR, promoting HIF-1α translation and enhancing the DNA-binding ability of HIF-1 [136]. ATR inhibition decreases HIF-1α expression and suppresses the expression of HIF-1-downstream genes, glucose transporter-1 (GLUT-1) and CAIX, under hypoxic conditions [136] and exhibits antitumor effect in in vivo studies with OE21 tumor xenograft, among others [60]. Inhibitors of ATR, such as AZD6738 and VX-970, are currently in phase I, II clinical trials as monotherapy and in combination with chemotherapy regimens, such as the PARP inhibitors Olaparib and Veliparib [137,138,139].

#### 2.3.4. Antitumor Effect of This Class of Drugs Inhibiting Translational Initiation of HIF-1α

The indirect inhibitory effects of Rapamycin, Cetuximab, Buparlisib, 2-MEs, Di-goxin, AZD6738, and VX-970 on translational initiation of HIF-1α certainly contribute to each of their antitumor activities. However, it is true that direct inhibitory effects of each drug on its target gene are also responsible for the antitumor activities.

### 2.4. Inhibitors of Stabilization of the HIF-1α Protein

#### 2.4.1. Drugs Increasing PHD2/VHL Activity

The stability of HIF-1α is mainly regulated through the actions of PHD2 and VHL. Briefly, PHD2 hydroxylates HIF-1α at two proline residues in the ODD domain, which triggers ubiquitination by VHL-containing E3 ubiquitin ligase and subsequent degradation by the 26S proteasome system. Therefore, drugs that potentially activate the PHDs/VHL/26S proteasome system have been expected to work as HIF-1 inhibitors.

- Melatonin and its derivative, NB-5-MT

Melatonin (also known as N-acetyl-5-methopxytryptamine) is a hormone that is secreted by the pineal gland of the brain in response to the day and night cycles for the regulation of the circadian rhythm. Recent studies have demonstrated that melatonin and its derivatives, such as N-butyryl-5-methoxytryptamine (NB-5-MT), have potent HIF-1α targeting activity through multiple molecular mechanisms [61,62]. Melatonin and NB-5-MT were found to activate PHDs’ activity, facilitating the interaction of HIF-1α protein with pVHL for its proteolysis [61,62]. In addition, melatonin increased VHL activity by suppressing the Akt/glycogen synthase kinase-3β (GSK-3β) signaling pathway, and eventually decreased HIF-1α protein stability [63]. Moreover, melatonin was reported to inhibit the expression of the HIF-1α protein by impairing HIF-1α protein translation [64]. The HIF-1 inhibitory activity of melatonin and NB-5-MT has been confirmed to significantly inhibit angiogenesis, epithelial-mesenchymal transition (EMT), and tumor growth in various mouse models with xenografted tumors of breast, prostate, and kidney cancer cells [62,65,66]. In vivo experiments using a zebrafish xenograft model with a colorectal cancer-derived cell line, HCT116, and, a triple negative breast cancer-derived cell line, MDA-MB-231, also demonstrated that NB-5-MT significantly inhibited tumor angiogenesis and delayed tumor growth [61,62]. Although NB-5-MT has not yet undergone clinical trial, antitumor activity of melatonin has already been confirmed in various cancer types.

- LW6

Treatment of cancer cells with LW6 induces VHL expression, leading to the degradation of the HIF-1α protein [67]. The action of LW6 was demonstrated to depend on the hydroxylation of the two proline residues in the ODD domain of HIF-1α, indicating that the inhibitor works by modulating the PHDs/VHL pathway. In a xenografted tumor model with human colon cancer cells, LW6 treatment was found to significantly delay tumor growth without causing severe side effects in the HCT116 xenografted tumor model [67]. There are currently no clinical trials being performed on LW6, but the preclinical data suggest that it may be an effective anticancer agent.

- GYY4137

Hydrogen sulfide (H_2_S) is a gasotransmitter involved in a wide range of biological processes [140]. Interestingly, H_2_S was recently reported to affect HIF-1α stability. Persulfidation of PHD2 at cysteine residues located in its zinc finger domain augments the interaction between PHD2 and HIF-1α, thereby promoting HIF-1α hydroxylation and degradation [68]. Treatment with GYY4137, a slow-releasing H_2_S donor, was shown to partially reverse the hypoxia-induced increase in HIF-1α protein levels and was also found to have anticancer effects in several cancer cell lines [68,69]. Experiments in mouse xenograft models showed that GYY4137 decreased tumor sizes without affecting the body weight or behavior of mice [69]. The therapeutic effect was reported to be attributed not only to the inhibitory effect on HIF-1 but also to the pro-apoptotic effect of GYY4137 [69]. Taken together, these findings suggest that GYY4137 may be a promising therapeutic in the future regardless of whether its mechanisms of action depend on HIF-1.

#### 2.4.2. Inhibitors Mediating HIF-1α Stability through HSP90

It has been repeatedly reported that the stability of the HIF-1α protein can be regulated independently of oxygen availability and the PHDs/VHL axis. For example, the receptor for activated C kinase 1 (RACK1) binds to the PAS-A domain of HIF-1α and promotes the ubiquitination and degradation of HIF-1α protein by facilitating recruitment of the Elongin C/B ubiquitin ligase complex [33]. This pathway is inhibited through the action of heat shock protein 90 (HSP90), an ATP-dependent molecular chaperone that aids in the correct folding, localization, and function of its client proteins [141,142]. Mechanistically, HSP90 interacts with HIF-1α at the PAS-A domain, competitively preventing RACK1 from binding, and thereby protecting HIF-1α from being tagged for proteasomal degradation [33,143]. Currently, there are several drugs that induce HIF-1α destabilization through the inhibition of HSP90.

- Ganetespib

Ganetespib is a small-molecule drug that binds to the ATP-binding pocket of HSP90 [70]. Experiments using colorectal cancer cell lines have shown that ganetespib treatment decreased HIF-1 protein expression, leading to a reduction in angiogenesis [71]. Moreover, Ganetespib treatment markedly impaired primary tumor growth and suppressed local tumor invasion and distant tumor metastasis to regional lymph nodes and lungs in orthotopic MDA-MB-231 and MDA-MB-435 tumor models [72]. Phase II clinical trials in patients with non-small cell lung cancer showed that ganetespib is able to elicit a clinical response without significant toxicity [73].

- Apigenin

Yet another HSP90 inhibitor is apigenin (4,5,7-trihydroxyflavone), a non-toxic dietary flavonoid abundantly found in various plants, such as celery, parsley, and chamomile [74]. Apigenin functions by inhibiting the kinase activity of casein kinase 2 (CK2), which decreases the phosphorylation of the HSP90 co-chaperone protein, cell division cycle 37 (Cdc37) [75]. This weakens the interaction between HSP90 and its target proteins, making them more susceptible to degradation [75]. In vitro experiments in various cancer cell lines and animal models with PC-3 and OVCAR3 showed that treatment with apigenin resulted in a reduction of angiogenesis and a decrease in the levels of the HIF-1α protein and its mRNA [75,76,77,78]. A phase II clinical trial investigating dietary supplementation of apigenin for the prevention of cancer recurrence was initiated, but was terminated without the release of the results. The promising experimental data and established safety of apigenin make it an attractive therapeutic option, but its efficacy in treating cancer remains to be demonstrated.

- Lonafarnib

Lonafarnib (SCH66336) is a farnesyltransferase inhibitor that has been shown to inhibit the interaction between HSP90 and HIF-1α [144]. In aerodigestive tract and endothelial cells, lonafarnib treatment decreased pro-angiogenic activity by decreasing the protein levels of HIF-1α and vascular endothelial growth factor (VEGF) [144]. Lonafarnib alone had a modest ability to inhibit the growth of orthotopic U87 tumors, but it significantly enhanced the therapeutic effect of the combination of radiation and temozolomide [79]. Phase I and II clinical trials administering lonafarnib alone or in combination with other drugs have shown that it is well tolerated and has anticancer activity mainly through the expected inhibitory effect on HIF-1 [80,81,82]. Assessment of lonafarnib’s utility as an anticancer agent has progressed to phase III clinical trials, although all trials have been prematurely terminated to date [83].

#### 2.4.3. Inhibitors Mediating HIF-1α Stability through Histone Deacetylases (HDACs)

HDACs are a group of enzymes that remove acetyl groups from histones and other proteins. It has been reported that HDACs can influence HIF-1 stability and activity through a wide variety of mechanisms. For example, they have been reported to stabilize the HIF-1α protein [88,145] and promote HIF-1α nuclear localization [84]. In general, HDACs are considered to function by modulating the acetylation status of either the HIF-1 protein itself or of cofactors such as p300 or HSP90 [84,88]. Given that HDACs are considered to regulate HIF-1 at multiple levels, drugs inhibiting HDACs may be potent HIF-1 inhibitors.

- Vorinostat (SAHA)

A well-known class I/IIb/IV HDAC inhibitor is vorinostat (suberoylanilide hydroxamic acid, SAHA). The exact mechanisms behind vorinostat’s inhibitory effects on HIF-1 are likely complex and remain to be fully characterized. Some authors have shown that vorinostat can reduce HIF-1α stability and suppress nuclear translocation by causing an increase in the acetylation of HIF-1’s chaperone, HSP90 [84]. In vitro and in vivo vorinostat treatment results in decreased cell growth, increased apoptosis, and reduced tumor size in a Hep3B tumor model [84,85,86]. This was accompanied by decreases in the levels of HIF-1α and VEGF proteins [84,85]. Vorinostat has been approved by the FDA for the treatment of cutaneous T-cell lymphoma (CTCL), and numerous phase I and II trials are in progress for the treatment of other types of cancer.

- Romidepsin (FK228)

Another class I/II HDAC inhibitor is romidepsin (FK228). Administering romidepsin to cells inhibits angiogenesis by sequentially reducing the expressions of HIF-1α and its downstream gene, VEGF, as well as suppressing DNA binding activity of HIF-1 [87]. Similar to vorinostat, romidepsin has been approved by the FDA for the treatment of CTCL. Romidepsin blocked angiogenesis in the Lewis lung carcinoma tumor model [87]. Phase I/II trials are being performed involving other types of cancer to evaluate the efficacy of romidepsin treatment alone as well as in combination with other therapies.

- Trichostatin (TSA)

TSA is a class I/II HDAC inhibitor. In cancer cells, it was reported to decrease cell proliferation and invasion, reduce angiogenesis, block cell cycle progression, and also induce apoptosis [89]. Treatment with TSA resulted in a reduction of both HIF-1α and VEGF protein levels [88,89]. Schoepflin et al. reported that the treatment of PHD2-null cells with TSA did not lead to any changes in HIF-1α levels [88]. Moreover, another study found that TSA increased VHL protein levels [90], indicating that TSA likely functions through targeting the canonical PHD/VHL axis. The safety and efficacy of TSA for the treatment of cancer patients have not yet been fully evaluated, but phase I clinical trials are in progress (NCT03838926).

#### 2.4.4. Antitumor Effect of This Class of Drugs Inhibiting Stabilization of the HIF-1α Protein

Among this class of drugs, the antitumor effects of LW6 and lonafarnib are thought to be dependent on their destabilizing effect on the HIF-1α protein because no other targets have been identified to date. On the other hand, the antitumor effects of the other drugs are thought to originate not only from their indirect suppressive effect on the HIF-1α protein but also from their inhibitory effect on each of their target pathways.

### 2.5. Inhibitor of HIF-1 Dimerization

- Acriflavine

HIF-1α and HIF-1β proteins harbor both a PAS domain containing PAS-A and PAS-B and a bHLH domain in each of their N-terminus regions. Heterodimer formation between HIF-1α and HIF-1β subunits mediated by these domains is required for the recruitment of HIF-1 to the HRE sequence in HIF-1’s target gene loci. Acriflavine, a mixture of 3,6-diamino-10-methylacridnium chloride (trypaflavine) and 3,6-diaminoacridine (proflavine), binds to the PAS-B domain of HIF-1α [91,92] and thereby prevents HIF-1 dimerization and inhibits transcription of HIF-1’s target genes, leading to antitumor effects without obvious adverse side effects in PC-3 xenograft tumor models. Although acriflavine is in a phase IV clinical trial for patients with recurrent cystitis [93] and has not been applied for cancer patients as yet, it is expected to be an effective drug for cancer therapy as well.

### 2.6. Inhibitors of HIF-1 DNA Binding

HIF-1 binds to the core DNA sequence (5′-R(A/G)CGTG-3′) in the HRE of target gene loci and activates their transcription. Therefore, inhibitors that target DNA binding activity of HIF-1 may be attractive drugs to improve cancer therapy.

- Echinomycin

Echinomycin (NSC-13502) was identified as a potent inhibitor of HIF-1’s DNA binding activity by a screening experiment [94]. The compound works as an intercalator for DNA in a sequence-specific manner and blocks the binding of HIF-1 to the HRE sequence. Treatment with echinomycin resulted in a reduction of VEGF mRNA expression in a dose-dependent manner in U251 cells [94]. Phase II clinical trials on echinomycin have been performed in patients with metastatic disease, but no significant clinical responses were observed in a phase II study [95,96]. Modulation of the echinomycin structure could be a beneficial approach to improve the therapeutic effect [97]. It has already been confirmed that liposomal-echinomycin provides significantly greater inhibition of primary tumor growth, and only liposome-formulated echinomycin can eliminate established triple-negative breast cancer (TNBC) metastases in a mouse model with MDA-MB-231 tumors [98].

- Anthracycline

Anthracycline chemotherapeutic drugs, including doxorubicin and daunorubicin, are extracted from *Streptomyces* bacteria and widely used for the treatment of cancer. Doxorubicin decreases HIF-1 transcriptional activity through the inhibition of HIF-1 heterodimer binding to the HRE sequences. Tanaka et al. showed that doxorubicin suppressed HIF-1-dependent cell migration in vitro and HIF-1 target gene expression in vivo [99].

- Radicicol

Radicicol is a heat shock protein inhibitor which binds to HSP90’s ATP-binding site [100]. Interestingly, it was reported that HIF-1 binding to HREs was reduced by radicicol treatment of cancer cell lines [101]. Both in vitro and in vivo treatment with radicicol resulted in a reduction in VEGF expression and angiogenesis [101]. These findings suggest that HSP90 may also play a role in maintaining the HIF-1 heterodimer in a conformation favorable to HRE binding. Radicicol has not been evaluated in clinical trials, but the experimental data so far suggest that it may exhibit favorable anticancer activity.

These three drugs exhibit an antitumor effect by inhibiting the recruitment of HIF-1 to HRE at least partly, but accumulating evidence suggests the importance of other mechanisms; echinomycin has been reported to inhibit myc oncogene [146], anthracycline (e.g., doxorubicin) induces death of cells through DNA-adduct formation, topoisomerase II inhibition, ROS production, etc. [147], and radicicol induces the degradation of various protein substrates by inhibiting HSP90 [100,101].

### 2.7. Inhibitors of HIF-1α Transactivation Activity

- Bortezomib

Bortezomib, a proteasome inhibitor, has been confirmed to repress the expression of HIF-1α protein and nuclear accumulation of HIF-1 by inhibiting the PI3K/Akt/mTOR and mitogen-activated protein kinase (MAPK) pathways, respectively [102]. In addition, bortezomib treatment suppresses HIF-1 transcriptional activity by inhibiting the interaction of p300 with HIF-1α [103]. Bortezomib exhibits favorable antitumor effect in preclinical mouse models with SiHa xenograft, which results from its inhibitory effects on the PI3K/Akt/mTOR and MAPK signaling pathways and HIF-1. The drug has been approved by the FDA for the treatment of multiple myeloma and mantle cell lymphoma. Further clinical trials have been conducted with bortezomib as monotherapy and combination therapy in the treatment of various other tumors [104,105].

### 2.8. Inhibitors of HIF-1α at Multiple Levels

- PX-478

The HIF-1α protein is known to be ubiquitinated by a pVHL-mediated mechanism for proteolysis under normoxic conditions. In addition, p53 promotes murine double minute2 (Mdm2)-mediated ubiquitination of the HIF-1α protein for degradation in an oxygen-independent manner [148,149,150]. PX-478 (S-2-amino-3-[4′-N,N,-bis(chloroethyl)amino]phenyl propionic acid N-oxide dihydrochloride) inhibits HIF-1α through multiple mechanisms, such as involving mRNA expression and translation levels. Moreover, PX478 has been reported to inhibit HIF-1α deubiquitination, resulting in a decrease of HIF-1α protein levels due to the accumulation of its polyubiquitinated form and antitumor effects in HT29 tumor xenografts [107,108]. Currently, phase I clinical trials are ongoing in patients with advanced solid tumors.

- Camptothecin and its analogues

DNA topoisomerase-I is inhibited by camptothecin, an alkaloid derived from *Camptotheca acuminate*, and its analogues including topotecan (TPT) and EZN-2208. These compounds are also known as potent inhibitors of HIF-1α translation, HIF-1α protein accumulation, and HIF-1 transcriptional activity [109,110,111]. TPT inhibits the translation of the HIF-1 protein independently of the oxygen environment, and the proteasome and the PI3K-Akt-mTOR pathways in vitro [110]. Furthermore, TOP administration suppresses tumor growth in xenograft models [112]. EZN-2208 also inhibits HIF-1α at protein levels and downregulates the mRNA and protein levels of HIF-1 target genes, such as VEGF, GLUT-1, and MMP-2 [111]. It is worth noting that both TPT and EZN-2208 strongly inhibit VEGF expression mediated by HIF-1 activity and lead to the suppression of pro-angiogenic activity in vivo [111,113].

- CRLX101

CRLX101, a nanoparticle conjugated with camptothecin, inhibits HIF-1α translation and HIF-1 activity. Recent preclinical studies reported that CRLX101 combined with bevacizumab, a humanized anti-VEGF monoclonal antibody, improved antitumor efficacy in murine models of tumor metastasis from an orthotopic triple negative breast cancer xenograft [114,115]. In a phase II clinical trial, CRLX101 in combination with bevacizumab was administered to patients with metastatic renal cell carcinoma (mRCC), and it showed that the combination protocol was safe [116]. However, a randomized phase II trial did not show a significant clinical improvement of progression-free survival in patients with mRCC [117].

- Metformin

Because metformin, a drug used to treat type 2 diabetes, decreases oxygen consumption in mitochondria by inhibiting mitochondrial complex I activity, it causes oxygenation of cytosol and induces the proteolysis of HIF-1α protein. In addition, metformin was reported to inhibit the expression of XBP1, which interacts with the HIF-1α protein and plays an important role in the transcription of HIF-1 downstream genes by facilitating the recruitment of RNA polymerase II [118,119,120]. Actually, metformin has been reported to decrease HIF-1 activity [118,119,120]. Metformin exhibits antitumor activity by suppressing mitochondrial respiration and HIF-1, as mentioned above. However, a phase III clinical trial with short-term treatment with standard diabetic doses of metformin did not reduce proliferation of endometrioid endometrial cancer [121].

PX-478 was originally found as an inhibitor of HIF-1, and no other target protein has been identified to date; therefore, antitumor activity of PX-478 is thought to be dependent on its suppressive effect on HIF-1. Because camptothecin (and its analogues) and CELX101 are known to inhibit topoisomerase-I activity and metformin inhibits mitochondrial respiration as described above, the antitumor effects of these drugs are thought to be attributed to these actions in addition to their inhibitory effects on HIF-1.

## 3. Conclusions and Perspectives

HIF-1, when activated by hypoxic or pseudo-hypoxic stimuli, or a genetic alteration of its regulators, promotes tumor growth by inducing the expression of its downstream genes related to angiogenesis, metabolic reprogramming, and invasion [1,2,3,4]. Moreover, HIF-1 is strongly associated with invasion, metastasis, and chemo-/radio-resistance of cancer cells [1,2,3,5,6,21,151,152]. For these reasons, HIF-1 is recognized as a rational target for cancer therapy, and actually, therapeutic effects of HIF-1 inhibitors have been repeatedly confirmed in both preclinical and clinical studies [1,2,6]. However, it is also true that HIF-1 inhibitors occasionally failed to exhibit favorable therapeutic effects in some studies. To overcome this problem and develop HIF-1-targeted drugs for clinical use, it is important to address some potential issues as described below.

Since HIF-1 is activated mainly in hypoxic regions of malignant solid tumors which are located approximately 70–100 μm from tumor blood vessels [4], it is difficult to deliver effective concentrations of chemotherapeutic agents there. Moreover, as HIF-1 is located in the nucleus as a transcription factor, it is not necessarily suitable as a therapeutic target in general. Therefore, it is thought necessary to optimize both pharmacokinetic and pharmacodynamic properties of HIF-1 inhibitors first in order to increase their activity.

As described in the Introduction, HIF-1 activity is regulated not only through the canonical PHDs-VHL-dependent mechanism but also through the non-canonical mechanisms at various regulatory steps. However, the molecular mechanisms behind them have not been fully understood yet. To develop an effective HIF-1 inhibitor and gain favorable therapeutic outcomes, it is critical to elucidate them in detail. It may also be important to seek a good combination of multiple HIF-1 inhibitors, each of which targets different regulatory steps.

HIF-1α has two isoforms, termed HIF-2α and HIF-3α, and the former shows high homology to HIF-1α at amino acid levels sharing 48% overall amino acid identity. Although HIF-2α functions as a tumor promoter exactly the same as HIF-1α, it has also been reported that it functions as a tumor suppressor as well in some specific cells. In this situation, it is important to elucidate the functional difference between the two isoforms and to identify cancer types where the specific isoform works suppressively in order to appropriately inhibit a specific isoform as needed. It is also necessary to develop an inhibitor specific to HIF-1 and to HIF-2.

Again, it is true that HIF-1 inhibitors did not necessarily exert antitumor activity in every preclinical and clinical study. This might have resulted, at least in part, from unsuitable research conditions, for example, the study that did not exclude patients with malignant tumors containing few hypoxic fractions or few HIF-1-positive cancer cells. To maximize the therapeutic benefit of HIF-1 inhibitors, we need to develop a companion diagnostic, in parallel, which enables the selection of patients to be treated with an HIF-1 inhibitor.

One of the important points we have to pay attention to during the development of HIF-1 inhibitors is that HIF-1 does not directly work in the initiation of malignant transformation and, therefore, is not simply categorized as a driver oncogene. Moreover, HIF-1 is not necessarily responsible for the survival of cancer cells. These are reasons why most HIF-1 inhibitors have the potential to delay tumor growth to some extent but cannot directly kill or eliminate cancer cells. Therefore, HIF-1 inhibitors are expected to synergistically gain a therapeutic effect by combining with other anticancer strategies. Recently, cancer therapy has entered an era of multidisciplinary treatment, and cancer patients are rarely treated with monotherapy. In this respect, it would be better to seek a therapeutic strategy to be combined with a HIF-1 inhibitor, which synergistically enhances the therapeutic effect of both during the development of the HIF-1 inhibitor.

The above issues are just examples, but they need to be preferentially addressed for the development of HIF-1 inhibitors with higher clinical efficacy in cancer therapy.

## Figures and Tables

**Table 1:** Potential HIF-1 inhibitors under development.

HIF-1 Inhibitors	Target Molecules	Tumors/Cell Lines
Transcriptional initiation		
Flavopiridol	blocking phosphorylation of RNA polymerase II’s COOH-terminal domain	acute myeloid leukemia, advanced renal cell carcinoma
Aminoflavone	AhR	breast cancer cells (MCF7), human renal cell lines
mRNA stabilization		
EZN-2968	HIF-1α mRNA	prostate (15PC3, PC3, and DU145), glioblastoma (U373) cells, duodenal neuroendocrine tumor
Translational initiation		
Rapamycin	mTOR	prostate cancer cells (PC-3)
Cetuximab	EGFR	HNSCC cells (FaDu, HN5, UMSCC1, and OSC19)
Buparlisib	PI3K	breast cancer
KC7F2	mTOR, S6K, 4EBP1	U251MG, MCF7, PC3, LNZ308, LN229 cells
2-MEs	tubulin	MCF7, MDA-MB-231, PC-3, ovarian cancer, multiple myeloma
Digoxin	Na^+^/K^+^ ATPase	Hep3B, 293, 293T, PC3, P493
AZD6738, VX-970	ATR	esophageal cancer cells (OE21, FLO-1) (VX-970)
Protein stabilization		
Melatonin, NB-5-MT	GSK-3β, activating PHDs	colon cancer cells (HCT116), prostate cancer cells (DU145, PC-3, LNCaP), breast cancer cells (MCF-7, MDA-MB-231), renal cell carcinoma (RenCa)
LW6	inducing pVHL expression	HCT116
GYY4137	slow-releasing H_2_S donor	HeLa, HCT-116, Hep G2, HL-60, MCF-7, MV4-11, U2OS
Ganetespib	ATP-binding site of HSP90	NSCLC (NCI-H1975, HCC827) cells, colorectal cancer (HCT116, HT29), non-small cell lung cancer
Apigenin	CK2 activity	multiple myeloma cells, pancreatic cancer cells (S2-013, CD18)
Lonafarnib	interaction between HSP90 and HIF-1α	non-small cell lung carcinoma, breast cancer
Vorinostat	increasing acetylation of HSP90	Hep3B, HepG2, U2OS, MG63, U87 MG, HuH7
Romidepsin (FK228)	HDAC	Lewis lung carcinoma (LLC), HT1080
Trichostatin	HDAC	tongue squamous cell carcinoma SCC-6, BAECs, 786-0 (VHL-negative), HEK 293 cells, Lewis lung carcinoma cells, HepG2
Heterodimerization		
Acriflavine	PAS-B domain of HIF-1α	HEK293, HeLa, Hep3B-c1, PC-3, P493
DNA binding		
Echinomycin	binding to HRE sequences	U251, MCF-7, non-small cell lung carcinoma, soft tissue sarcoma
Anthracycline	HIF-HRE binding	HK-2. HeLa, A498, KMRC-3 VHL-defective renal cell carcinoma (RCC) cells, Caki-1 VHL-competent renal cell carcinoma cells
Radicicol	HIF-DNA binding, HSP90	SKBR3, Hep 3B
Transactivation		
Bortezomib	PI3K/Akt/mTOR, MAPK, p300 recruitment	prostate cancer cells, Hep3B, HEK293, ARH77, U299 cells, RCC, breast cancer, rectal carcinoma, nasopharyngeal, neuroendocrine carcinoma, prostate cancer
Other steps		
PX-478	inhibiting HIF-1α deubiquitination, reducing HIF-1α mRNA and HIF-1α translation	PC-3, MCF-7, HT-29, Panc-1, BxPC-3 cells, RCC4, RCC4/VHL, HCT116
Camptothecin analogues (TPT, EZN-2208)	topoisomerase-1	HEK293, U251, leukemia cell lines, neuroblastoma cells (GI-LI-N, LAN-5) derived from metastatic
CRLX101	topoisomerase-1	ovarian cancer, breast cancer, renal cell carcinoma
Metformin	Mitochondria complex I, XBP1	HCT116, HCC, NSCLC, etc.

AF, aminoflavone; AhR, aryl hydrocarbon receptor; ATR, ataxia telangiectasia and Rad3-related kinase; CK2, casein kinase 2; EGFR, epidermal growth factor receptor; HDACs, histone deacetylases; HIF-1, hypoxia-inducible factor 1; HNSCC, head and neck squamous cell carcinoma; HRE, hypoxia response element; H_2_S, hydrogen sulfide; HSP90, heat shock protein 90; GSK-3β, glycogen synthase kinase-3β; MAPK, mitogen-activated protein kinase; 2-ME, 2-methoxyestradiol; RCC, renal cell carcinoma; mTOR, mammalian target of rapamycin; 4EBP1, eukaryotic translation initiation factor 4E-binding protein 1; PAS, Per-Arnt-Sim; PI3K, phosphatidylinositol-3-kinase; pVHL, von Hippel-Lindau protein; TPT, topotecan; TSA, trichostatin A; XBP1, X-box binding protein 1.

**Table 2:** Potential HIF-1 inhibitors under development.

HIF-1 Inhibitors	Animal Models	Clinical Trials	References
Transcriptional initiation			
Flavopiridol	No animal model looking at effect on hypoxia/HIF-1	Not in clinical use	[35,36,37,38,39]
Aminoflavone (AF)	MCF-7 xenografts	Phase 1 (Completed)	[40,41,42,43]
mRNA stabilization			
EZN-2968	DU145 xenograft	Phase 1 (Completed)	[44,45]
Translational initiation			
Rapamycin	LKB1 ± mice (Peutz-Jeghers syndrome model), ACHN xenograft, SN12C xenograft	FDA-approved for treatment for renal cell carcinoma (RCC)	[46,47,48]
Cetuximab	Tgfb1/Pten deletion 2cKO mice, CAL27 xenograft	FDA-approved for head and neck squamous cell carcinoma (HNSCC), colorectal cancer (CRC)	[49,50]
Buparlisib	No animal model looking at effect on hypoxia/HIF-1	Phase 3 (Completed, Terminated)	[51]
KC7F2	No animal model looking at effect on hypoxia/HIF-1	Not in clinical use	[52]
2-MEs	LLC xenograft/MDA-MB-231 xenograft	Phase 2 (Completed)	[53,54,55,56,57]
Digoxin	P493 xenograft/PC3 xenograft	Phase 2 (Completed, Terminated)	[58,59]
AZD6738, VX-970	OE21 xenograft (VX-970)	AZD6738: Phase 2 (In progress)	[60]
		VX-970: Phase 2 (In progress)	
Protein stabilization			
Melatonin, NB-5-MT	Tumor xenograft models with breast, kidney, prostate, and colon cancer cells in mouse and zebrafish	Not in clinical use	[61,62,63,64,65,66]
LW6	HCT116 xenograft	Not in clinical use	[67]
GYY4137	No animal model looking at effect on hypoxia/HIF-1	Not in clinical use	[68,69]
Ganetespib	Orthotopic implantation of MDA-MB-231, MDA-MB-435	Phase 2 (Completed) Phase 3 (Terminated)	[70,71,72,73]
Apigenin	PC-3 and OVCAR3 Matrigel plug assay	Phase 2 (Terminated)	[74,75,76,77,78]
Lonafarnib	Orthotopic implantation of UMSCC38	Phase 2 (Completed) Phase 3 (Terminated)	[79,80,81,82,83]
Vorinostat	Hep3B xenograft	FDA-approved for cutaneous T-cell lymphoma Phase 2 with results for other cancer types	[84,85,86]
Romidepsin (FK228)	Orthotopic implantation of LLC	FDA-approved for cutaneous T-cell lymphoma Phase 2 with results for other cancer types	[87]
Trichostatin	No animal model looking at effect on hypoxia/HIF-1	Phase 1 (In progress)	[88,89,90]
Heterodimerization			
Acriflavine	PC-3 xenografts	Not in clinical use	[91,92,93]
DNA binding			
Echinomycin	MDA-MB-231 and SUM-159 xenografts	Phase 2 (Completed)	[94,95,96,97,98]
Anthracycline	HeLa xenografts	Phase 2 (Completed)	[99]
Radicicol	No animal model looking at effect on hypoxia/HIF-1	Not in clinical use	[100,101]
Transactivation			
Bortezomib	SiHa xenografts	Phase 1 (Completed)	[102,103,104,105,106]
Other steps			
PX-478	HT-29 xenografts	Phase 1 (Completed)	[107,108]
Camptothecin analogues (TPT, EZN-2208)	U251 glioma xenografts	TPT: Phase 1 (Completed)	[109,110,111,112,113]
		EZN-2208 combination with other drugs: Phase 1 (Completed)	
CRLX101	orthotopic primary triple-negative breast cancer xenografts	Phase 2 (Completed)	[114,115,116,117]
Metformin	Various types of tumors, e.g., CRC and HCC	Phase 3 (Completed)	[118,119,120,121]

CTCL, cutaneous T-cell lymphoma.
